# A 20-Year Systematic Review of the ‘Reading the Mind in the Eyes’ Test across Neurodegenerative Conditions

**DOI:** 10.3390/brainsci13091268

**Published:** 2023-08-31

**Authors:** Owen Stafford, Christina Gleeson, Ciara Egan, Conall Tunney, Brendan Rooney, Fiadhnait O’Keeffe, Garret McDermott, Simon Baron-Cohen, Tom Burke

**Affiliations:** 1 School of Psychology, University College Dublin, D04 F6X4 Dublin, Ireland; 2 School of Psychology, University of Galway, H91 TK33 Galway, Ireland; 3 Acquired Brain Injury Ireland, Meath Services, Dublin, Ireland; 4 St Vincent’s University Hospital, D04 T6F4 Dublin, Ireland; 5 Department of Psychology, Tallaght University Hospital, D24 NR0A Dublin, Ireland; 6 Autism Research Centre, Department of Psychiatry, Cambridge University, Cambridge CB2 8AH, UK; 7 Centre for Neuroimaging, Cognition, and Genomics，University of Galway, H91 TK33 Galway, Ireland

**Keywords:** social cognition, neurodegenerative diseases, the ‘Reading the Mind in the Eyes’ test, Huntington’s disease, motor neuron disease, multiple sclerosis, Lewy body dementia, Alzheimer’s disease, Parkinson’s disease

## Abstract

Social cognition has a broad theoretical definition, which includes the ability to mentalise, i.e., recognise and infer mental states to explain and predict another’s behaviour. There is growing recognition of the clinical, diagnostic, and prognostic value of assessing a person’s ability to perform social cognitive tasks, particularly aspects of theory of mind, such as mentalising. One such measure of mentalising is the ‘Reading the Mind in the Eyes’ test (RMET). This systematic review and meta-analysis consider performance on the RMET, applied to people with neurodegenerative conditions in matched control studies, since its publication in 2001. Overall, this review includes 22 papers with data from N = 800 participants with neurodegenerative conditions: Alzheimer’s disease, *n* = 31; Parkinson’s disease, *n* = 221; Lewy body dementia, *n* = 33; motor neuron disease, *n* = 218; Huntington’s disease *n* = 80; multiple sclerosis, *n* = 217; and N = 601 matched typical controls. Our meta-analyses show that deficits in mentalising, as measured by the RMET, are consistently reported across neurodegenerative conditions, with participants in both early and late disease stages being affected. Social cognition is an emerging field of cognitive neuroscience requiring specific and sensitive measurement across each subdomain. Adult-based meta-normative data feature, for which future groups or individuals could be compared against, and hypotheses relating to the source of these mentalising deficits are further discussed. This review was registered with PROSPERO (CRD42020182874).

## 1. Introduction

Social cognition refers to a variety of behaviours and cognitive processes which underpin how we understand, interpret, and interact with social contexts and others [1,2]. The ‘social brain’ includes anatomical networks associated with frontal (e.g., prefrontal cortex (PFC)) and temporal lobes (e.g., temporoparietal junction (TPJ)) [2]. Defects within these anatomical regions may lead to impairments, specifically in the domain of social cognitive functions, i.e., emotion recognition and theory of mind (ToM). Taken together, both are proximal predictors of social functioning and are associated with reduced quality of life across neurodegenerative and neurodevelopmental conditions [3,4]. Conceptually, emotion recognition and ToM are related in part; however, a distinction exists between the low-level perceptual processing and decoding of affective states involving emotion recognition and the higher-level cognitive process of mental state deduction associated with ToM [5]. Research and meta-analyses involving neurodegenerative, psychiatric, and developmental populations have previously analysed emotion recognition and ToM as separate concepts, yielding varying results across groups [6]. For example, Altschuler and colleagues [7] found that affective ToM difficulties may relate to face processing in autism spectrum disorder (ASD) but not in schizophrenia spectrum disorder (SCZ) or among those in the typical development (TD) groups.

ToM is an overarching term for a complex series of cognitive functions typically dichotomised into the Cognitive Theory of Mind (CToM) and Affective Theory of Mind (AToM). The CToM conceptually enables one to accurately interpret and/or predict social context, social appropriateness, and social cues and is associated with dorsolateral frontostriatal circuitry activity [8,9,10]. Conversely, AToM typically relates to the ability to mentalise or infer another person’s feelings, preferences, and/or mental state and is largely associated with the orbitofrontal cortex involving the orbital–frontostriatal circuitry state [3,11].

Due to the vast anatomical regions implicated in social cognitive processes, impaired social cognition is common in people with neurodegenerative conditions, and specifically where disruptions in prefrontal and orbito-frontostriatal regions are implicated in disease pathophysiology [1,8,12]. Social cognitive deficits have been identified across many neurodegenerative conditions, including Huntington’s disease (HD), multiple sclerosis (MS), amyotrophic lateral sclerosis (ALS), and behavioural variant frontotemporal Dementia (bvFTD), where cortical disruptions impacting the striatum and prefrontal cortex are evident [13,14,15,16]. Social cognitive impairment among neurodegenerative populations rarely occurs in isolation, resulting from network-based degeneration and consequent executively based cognitive impairment. By way of example, in advanced Parkinson’s disease (PD), interactions between the ventromedial prefrontal cortex (vmPFC) and the amygdala result in frontostriatal–limbic-circuitry dysfunction, leading to impaired decision making and decoding facial expressions [10,17,18]. Notwithstanding, in late stages of disease progression, a social cognitive deficit may be secondary to difficulties in non-social cognitive impairment, and as such, sensitive and specific measures of social cognitive deficits are becoming increasingly important in identifying deficits at an earlier stage of the disease development. Furthermore, reliable and sensitive measures of social cognitive deficits are central for discriminating between disease subtypes, predicting prognosis, and aiding with differential diagnostics [12].

The advancement regarding the the relevance and importance of assessing social cognition among clinical populations is highlighted in the current edition of the American Psychiatric Association’s Diagnostic and Statistical Manual for Mental Disorders (DSM-5), which includes social cognition as one of the six core neurocognitive domains. Despite a lack of recommendations for proprietary tests of social cognition included in the DSM-5, the need for objective quantification is necessary to assess the severity of structural and/or functional impairment, in addition to facilitating clinical decision making. Social cognition is broadly grouped across four domains: ToM, affective empathy, social perception, and social behaviour [19,20]. Each domain can be assessed using specific and validated measures, for example, the ‘Reading the Mind in the Eyes’ test (RMET), the Faux-Pas Test, and False-Belief Tasks are commonly used in mentalising and ToM measurement, respectively [21,22,23].

The RMET [21] is the focus of this review, and it is a widely used measure of mentalising, and previous reviews have found that it is commonly used to detect deficits in clinical populations [24,25]. It consists of 36 black-and-white pictures of a person’s ocular region and one practice item. The participant is required to recognize the mental state represented in the picture and to choose one of four force-choice responses presented. Each item is scored 1 if the answer is correct and 0 if the answer is incorrect, with a range of 0–36 on the measure. In addition to having good test–retest reliability, it is not confounded by gender when assessed on large-scale groups, and it can be further stratified by positive, negative, or neutral stimuli valence, with 18-item repeatable short forms also available [24,25,26,27,28,29]. Pragmatically, the RMET may be particularly useful as a multimodal measure of mentalising among neurodegenerative populations, as it can be responded to verbally or by pointing and contains a glossary of terms should an individual be unfamiliar with a word.

This systematic review and meta-analysis aimed to investigate performance across neurodegenerative conditions over the past 20 years which report the use of the RMET alongside a cohort of healthy/typical controls, since its development in 2001 [21]. A further aim of this study was to extract and collate the normative data reported through the typical control (TC) cohorts and create a set of multinational, multisite, typical comparative performance data for the RMET which may support normative benchmarking for future evaluations using this tool, on both an individual and group level. 

## 2. Methods

The Preferred Reporting Items for Systematic Reviews and Meta-Analysis (PRISMA) guidelines [30] were followed. This systematic review and meta-analysis were formally registered with PROSPERO (CRD42020182874) prior to beginning the searches and data extraction. 

### 2.1. Search Strategy

A systematic literature search of the PubMed, Medline, and PsycINFO databases was carried out in the period extending from February to December 2020. This search strategy included iterative processes, using a combination of keywords, index terms, Boolean Operators, and search strings. Search terms included ‘theory of mind’ OR ‘mentalising’ OR ‘social cognition’, in combination with the required subfield of ‘Reading the Mind in the Eyes’. The latter search criteria were used specifically, as any study using the version of the RMET required for this review would have referenced the original measure [21], regardless of the referencing style of the associated publication, and, consequently, this search term would appear in the reference list. Articles were screened for inclusion from the reference lists of relevant articles, and no language restriction was imposed at this stage of the search process. The grey literature (case reports, conference abstracts, theses, reviews, and editorials) was considered and subjected to the predefined inclusion criteria for this review to reduce potential publication bias [31]. The time restriction placed on the literature search was between January 2001 and December 2020 inclusive. The 36-item version of the RMET, published in 2001, was the subject of this review. The final review search of the 2020 literature was conducted in September 2021 to ensure late-published 2020 material was appropriately reviewed and included where relevant. 

### 2.2. Inclusion Criteria

Only papers published in English were included for full review. Potentially included studies had their methods section reviewed to ensure that the English-version of the tool was used. Studies that reported objective quantitative investigations using the full 36-item version of the RMET were included. In the initial searches, all clinical groups with matched TCs were required, with neurodegenerative conditions extracted specifically for the primary purpose of this review, and TC data were used as meta-normative data. 

Specifically, studies were required to have a TC comparative group and report-outcome data for the RMET. RMET raw data, percentage correct, or standardised scores were deemed acceptable, and converted for uniform reporting. Studies which presented a sub-cohort of individuals with a neurodegenerative condition as part of a larger group study or cohort, were included if the patient group was clearly demarcated within the original study. 

### 2.3. Exclusion Criteria

Mixed sample studies, where people with a neurodegenerative condition were grouped together, were excluded, unless, as stated above, a subsample was identifiable for the individualised cohorts and the data were available accordingly. Qualitative studies and non-assessment-based studies of the RMET were not considered. Studies which only contained subjective self-report cognitive outcomes were not considered for the purpose of the review, unless accompanied by objective RMET assessment. Studies which used any version of the RMET other than the original [21] version were excluded, e.g., shortened versions, versions with novel stimuli included in replacement of the original, non-English-language versions, and any adapted or non-standardised versions of the test. Studies without a TC cohort comparison were excluded. Studies pertaining to Mild Cognitive Impairment (MCI) were also excluded due to the uncertainty regarding progression to a neurodegenerative condition, as well as the heterogeneous MCI presentation. 

### 2.4. Study Selection 

The titles and abstracts of the articles were independently screened for relevance by two reviewers (OS and TB); relevant publications, potentially eligible for inclusion, were read in full text by two reviewers (OS and TB). Specifically, the version and properties of the RMET used within each study were further reviewed by both reviewers to confirm inclusion. 

### 2.5. Risk-of-Bias Assessment and Quality of Evidence

The risk of bias of the included studies was assessed independently by the two researchers (OS and TB), who assessed the suitability of the full-text articles. These studies were assessed for methodological quality, using a published checklist by Hawker, Payne, Kerr, Hardey, and Powell [32] that identifies nine specific elements of the published report, each evaluated using a 4-point Likert-type scale (4 = good, 3 = fair, 2 = poor, 1 = very poor). The 9 elements included: (1) the abstract and title, (2) introduction and aims, (3) method and data, (4) sampling procedure, (5) data analysis, (6) ethical consideration and approvals, (7) findings and results, (8) transferability/generalizability, and (9) implications and usefulness. Scores for each article were summed with higher scores reflecting higher methodological quality (scores range from 9 (very poor) to 36 (very good). The first author assessed the quality of each study retained in the search and found that most of the studies were of good methodological quality, with scores ranging from 26 to 36. A second author (TB) randomly selected 30% of the studies and conducted an independent assessment. No significant disagreements occurred, and an inclusion consensus was achieved.

The Grading of Recommendations Assessment Development and Evaluation (GRADE) approach was further used to assess the overall quality of evidence for each variable considered within this study [33]. Two researchers (OS and TB) rated the factors on the GRADE criteria. The criteria ‘dose effect’, ‘inconsistency’, and ‘moderate/large effect’ were omitted, as these criteria were not applicable to the quality of evidence for the present study.

### 2.6. Data Synthesis

Data were independently extracted from eligible papers, using structured data forms that were developed specifically for this study, and these included key components of the study characteristics, study results, and methodological quality of the studies. RMET performance data were extracted as the primary outcome measures for this review (M ± SD; correct score), with impairment or between-group statistical differences noted, per study. Data were requested directly from authors, if not available and/or reported specifically. 

The GRADE criteria allowed for the quantification of potential publication bias. Study authors were contacted for clarification, supplemental demographic data, and/or outcome data as required. Standardised functional and clinical outcome scales of each neurodegenerative condition were considered, if included in the original study. 

### 2.7. Meta-Analysis

Meta-analyses were conducted using Review Manager V5.4 to analyse and present an overall effect estimate for the RMET when comparing individual studies of neurodegenerative groups compared to TCs, using a fixed-effects model. Analyses were performed between the individual studies, as well as diagnostic subgroups relative to the TCs. In instances of high heterogeneity in these data, a random-effects model was employed. An examination of interstudy heterogeneity was conducted using both the χ² and I² statistics. According to the Cochrane Handbook for Systematic Reviews of Interventions [34], indices of under 30% are considered to be low, 30–50% to be moderate, 50–75% to be substantial, and 75–100% to be very high. Funnel plots were created, where relevant, to determine the relative risk of publication bias in the sample of included studies.

## 3. Results

### 3.1. Studies Selected 

**Figure 1:**
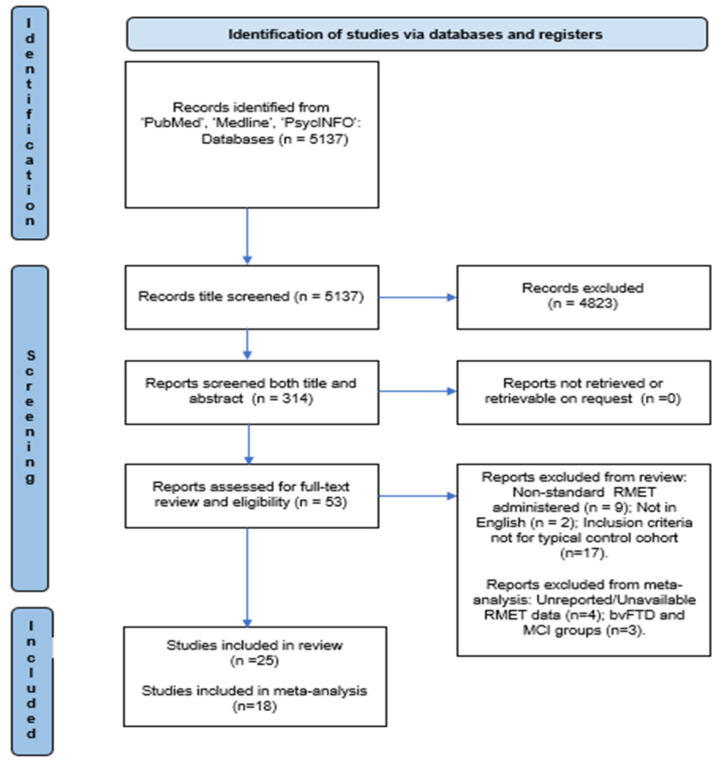
PRISMA flow diagram of studies included in this systematic review.

The search identified a total of 53 potentially relevant articles following title and abstract screening. After the removal of duplicates and full-text screening, a total of 22 studies were included (see Figure 1). This yielded a total of *n* = 800 participants and *n* = 601 matched TCs. Within the cohort of people with a neurodegenerative condition, the breakdown is as follows: Alzheimer’s disease (AD), *n* = 31; Parkinson’s disease (PD), *n* = 221; Lewy body dementia (DLB), *n* = 33; motor neuron diseases (MNDs), *n* = 218; Huntington’s disease (HD), *n* = 80; and multiple sclerosis (MS), *n* = 217. 

### 3.2. Description of Studies Included

The key characteristics of each study are presented in Table 1. The included studies were published in 10 different countries, and the publication date ranged from 2010 to 2020, despite the included timeline for the review being from 2001 to 2020. Among these, 20 studies were cross-sectional, and 2 were longitudinal studies. Individual study samples ranged from 12 to 106 patients and 12 to 65 TCs. The mean age of patients ranged from 34.2 to 78.1 years, with the time from diagnosis to assessment ranging from 10.8 months to 47 months within the cross-sectional studies, where reported. Individual study data can be seen in Table 1.

### 3.3. Risk of Bias and Quality Evidence

Table 2 reports the overall quality of the studies included within this review, as well as the individual breakdown of performance on the aforementioned criteria. Regarding the GRADE approach, there were no serious limitations (≥75% agreement between studies) in relation to study limitations, indirectness, and publication bias. Potential publication bias was observed for the studies reporting outcomes for people with AD and DLB, as each condition has ≤3 publications included within the systematic review and meta-analysis.

### 3.4. RMET Performance 

#### Meta-Analysis and Typical Control Outcomes

**Figure 2:**
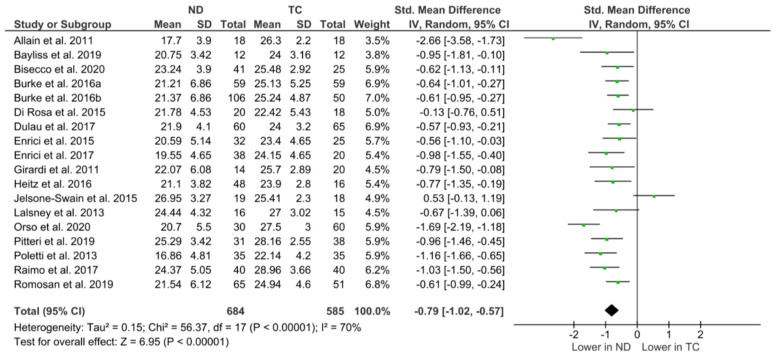
Forest plot of effect sizes with their 95% confidence intervals (CIs), and respective weights are shown for each individual study on RMET when compared with typical controls (TCs). The average effects of all studies combined is represented by the black diamond at the bottom of the graph. Due to high heterogeneity within this comparison, effect sizes are represented here as a standardised mean difference (SMD) [14,15,16,35,36,37,38,39,40,41,42,43,44,45,46,47,48].

For the overall evaluation of the mentalising performance between the combined neurodegenerative groups and TCs, 18 studies were included in the meta-analysis [14,15,16,18,35,36,38,39,41,42,43,44,45,46,48,49,50,51], with 4 studies excluded due to unreported and/or unavailable RMET data [10,37,40,47]. Figure 2 illustrates the random-effects meta-analysis, which yielded a very large overall effect size (SMD, −0.79; 95% CI, −1.02 to −0.57; *p* < 0.001), with high levels of heterogeneity across studies (χ² = 291.64; I² = 93%).

**Figure 3:**
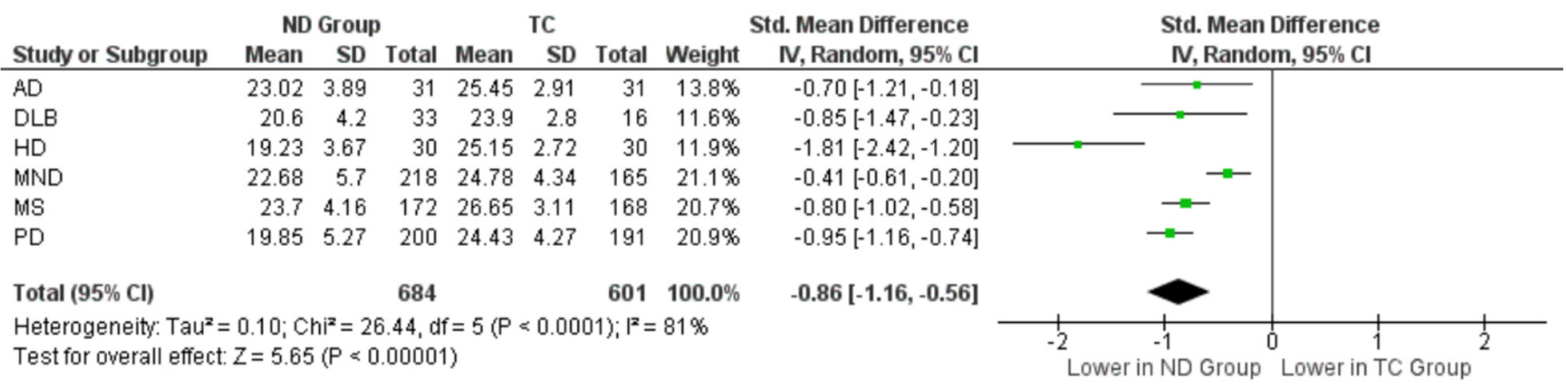
Forest plot of effect sizes, their 95% confidence intervals, and the respective weight of each neurodegenerative category (ND) on RMET performance as compared with typical controls (TCs). The average effects of all studies combined is represented by the black diamond at the bottom of the graph. Due to high heterogeneity within this comparison, effect sizes are represented here as a standardised mean difference (SMD). See Supplementary Material one for overview of neurodegenerative disease characteristics: Alzheimer’s Disease (AD); Dementia with Lewy Body (DLB); Huntington’s Disease (HD); Motor Neurone Disease (MND); Multiple Sclerosis (MS); Parkinson’s Disease (PD).

The individual studies (reported in Figure 2) were then grouped by diagnosis; see Figure 3, which further illustrates a significant deficit in RMET performance across all groups, i.e., AD, DLB, HD, MS, MND, and PD cohorts, compared to their respective TC cohort (*p* < 0.05, respectively). The most profound effect is shown in the HD group (SMD, −1.81; 95% CI, −2.42 to −1.20; *p* < 0.05).

As each study within the meta-analysis uses the RMET with a TC cohort, it is possible to create meta-based norms for the task. The TC cohort is derived from participants within Australia, France, Germany, Ireland, Italy, Romania, Scotland, Spain, the UK, and the USA. Within the TC cohort for the meta-norms, 53.47% were female, and the average age was 57.27 years ± 11.24, with 11.83 ± 2.35 years of education. On a group level, as investigated for the purpose of this review, the distribution of TCs scores were found to be normally distributed. Considering performance for this cohort, the mean raw score outcome and standard deviation was 25.20 ± 3.66 (average percentage correct: 70% ± 10.16%). Consequently, the range of raw scores would be considered in the following ways: Impaired ≤17; 18−21 Low Average; 22−28 Average; 29−32 High Average; ≥33 Superior.

### 3.5. Specific Outcomes across Neurodegenerative Cohorts

#### 3.5.1. Alzheimer’s Disease 

Of the studies investigating mentalising in those with AD and using the RMET included in this review (*n* = 2), variations in study findings were reported. Laisney et al. [36] observed mentalising deficits in participants with mild-to-moderate AD, with the AD group performing significantly worse (mean ± SD; 24.44 ± 4.32; *p* < 0.04) than age- and education-matched TCs (27 ± 3.02) on the RMET. Laisney et al. [36] also used a Judgement of Preference task, which, similar to the RMET, requires the decoding of mental states and for participants to identify the focus of other people’s attention based on the direction of their gaze. A significant group effect was also found on this measure with lower performances for the participants with AD (15.2 ± 6.4) relative to TCs (19.6 ± 0.9). In contrast, Heitz et al. [41] found no significant difference in performance on the RMET when participants with probable/early AD were compared to a TC group, and when AD scores were compared to a group of patients with dementia with Lewy bodies (DLB; described below).

The results of the meta-analysis showed that groups of participants with AD performed significantly worse on the RMET as compared with TCs, with a moderate effect size (SMD, −0.70; 95% CI, −1.21 to −0.18; *p* < 0.01). A further examination of heterogeneity showed that the two samples included in the subgroup analysis had a homogenous degree of variability (χ² = 0.01; I² = 0%). 

#### 3.5.2. Dementia with Lewy Bodies (DLB)

As noted above, Heitz and colleagues [41] reported outcomes between AD, DLB, and TCs. When comparing participants with DLB (*n* = 33) to TCs (*n* = 16), there was no significant difference in demographics, though the TC group performed significantly better on the Mini-Mental State Examination (MMSE; 29.3 ± 0.9 vs. 27.2 ± 1.8; *p* < 0.001). Furthermore, there was a statistically significant difference between the TC and DLB group performance on the RMET (23.9 ± 2.8 vs. 20.6 ± 4.2; *p* = 0.015, respectively). As mentioned above, the AD group in this study did not statistically differ from TCs. The Social Faux Pas test was also used as part of the mini-SEA battery, with a similar pattern observed for participants with DLB performing worse than controls (*p* = 0.033), but no difference was observed between AD and controls or DLB. Heitz et al. [41] further investigated the atrophy of grey matter, using voxel-based morphometry in an exploratory whole-brain analysis sequence. Their findings indicated that impairment on the RMET in the DLB group was correlated with diminished volume of the right middle and inferior frontal gyri (*p* < 0.001).

As there is only one study on the RMET and DLB which met the inclusion criteria for this systematic review, the overall outcomes should be interpreted with caution, as they are at risk of publication bias. For this reason, DLB could not be included in the disease specific meta-analysis (Figure 3); however, one study [41] is illustrated in Figure 2 relative to the other studies. 

#### 3.5.3. Huntington’s Disease (HD)

Of the studies relating to participants with HD included in this review (*n* = 4), one study [49] reported the performance on the RMET by using the median and interquartile range, thus requiring scores to be converted to represent the mean and standard deviations for both the experimental and control groups.

Allain et al. [35] investigated whether participants with HD had lower cognitive and/or affective ToM abilities relative to TCs. The RMET was used to assess mentalising, whilst the Attribution of Intention (AI) task measured cognitive ToM. Participants with HD had a significantly lower performance on both the AI (*p* < 0.0001) and RMET (*p* < 0.0001) when compared to age- and education-matched TCs. The findings also revealed that participants with HD performed significantly lower than TCs on tasks of executive functioning, with low scores on the RMET associated with low scores on verbal fluency (*p* = 0.01) and response inhibition (*p* = 0.01), as measured using the Stroop test.

Eddy et al. [37] found that, in addition to participants with HD making significantly more errors than TCs on the RMET, they also exhibited significant impairments on measures of executive function, including verbal fluency tasks and the Trail-Making Tests. The HD group in this study was stratified by either motor or psychiatric symptomatology. The Stroop test was also employed, which, contrary to the findings of Allain et al. [35], found no between-group significance (*p* = 0.084). A correlation analysis explored the relationship between RMET performance and the Social Faux Pas test recognition score and found significant positive correlations (*r* = 0.414; *p* = 0.023), as did correlations with a task of spatial perspective taking (*r* = 0.393; *p* = 0.032). Similar to the findings of Allain et al. [35], there was a significant negative correlation between the RMET and the error rates on both verbal fluency (*r* = −0.642; *p* = 0.001) and semantic fluency (*r* = −0.694; *p* = 0.001).

Eddy and Rickards [40] examined premanifest gene-positive participants with HD. Despite the premanifest HD group displaying intact executive skills, significant between-group differences were found on measures of cognitive and affective ToM. More specifically, 80% of participants with premanifest HD made more errors on the RMET than the average control (*p* < 0.01). Semantic fluency revealed a significant group difference, which related to the ToM performance in that poorer fluency scores predicted 73% of the variance in performance on the RMET. 

Bayliss et al. [49] compared participants with mild-to-moderate HD to their relatives (spouse or at-risk first-degree relative with negative gene tests) and TCs, with results indicating that performances on the RMET were significantly lower in the HD group than for those of the HD relatives and TCs (*p*= 0.016). Participants with mild-to-moderate HD in this study did not perform significantly lower on the MoCA or the Pictures of Facial Affection when compared to the control cohorts. This further suggests that deficits in social cognition can occur in the absence of global or executive-specific decline and may represent an early marker of cognitive dysfunction in HD.

Overall, studies involving HD populations included in this review indicated a specific mentalising impairment considering a premanifest, mild, or moderate disease stage. Except for measures of fluency, which showed a consistent relationship with RMET performance in HD groups, mentalising deficits seemed likely to be present in the absence of executive dysfunctions and irrespective of executive impairments declining alongside disease progression. 

Two of the HD studies were included in the meta-analysis [35,49]. A random-effects analysis revealed that participants with HD performed significantly worse on the RMET than the TCs did (SMD, −1.81; 95% CI, −2.42 to −1.20; *p* < 0.001). However, the analysis also revealed a very high degree of heterogeneity within the sample (χ² = 7.06; I² = 86%). 

#### 3.5.4. Motor Neuron Disease 

The studies examining ALS populations included in this review (*n* = 3) represent the heterogeneous cognitive profiles of those living with this neurodegenerative condition. Girardi et al. [15] used the RMET as a measure of simple and complex emotional understanding and found that a sample of cognitively intact participants with ALS showed a specific ToM deficit, which negatively correlated with verbal fluency performance (*r* = −0.66; *p* < 0.05). There was no statistically significant difference in performance on the RMET when participants with ALS were compared to the TC group (*t* = 2.08; df = 17.2; *p* > 0.05). No significant relationship was reported between RMET performance and behavioural measures (i.e., Manchester Behavioural Questionnaire [52], Cambridge Behavioural Inventory [53], and Frontal Systems Behaviour Scale [54]). For the participants with ALS, when the Judgement of Preference task was administered to infer the mental state of another as determined by eye gaze, a significantly poorer performance was reported. 

Jelsone-Swain et al. [39] compared performance on the RMET between participants with ALS and TCs, in addition to a subset of participants with ALS, who performed at the ‘ceiling effect’ on an action-understanding task. It was found that those with greater performance in the action-understanding task also performed better on the RMET when compared to participants with ALS, who performed worse on the task (*p* < 0.05). While this difference may be due to differences within the group relative to uncharacterised cognitive phenotypes, a further fMRI analysis revealed that participants in the upper action-understanding-task group had greater activation in regions associated with social cognition and mentalising ability, namely the bilateral superior frontal gyrus. The performances on the RMET between participants with ALS (26.95 ± 3.27) and TCs (25.41 ± 2.3) were not significantly different. Of note, this is the only study within this review whereby the neurodegenerative cohort outperformed the TCs on the RMET. No between-group differences in verbal fluency (COWAT), general cognition (ALS-CBS and MoCA), or the RMET were observed following a MANCOVA with contrived education level (F(1,31) = 0.60, *p* = 0.71). 

Burke et al. [42] used normally distributed control data to compare a within-group sample of participants with ALS who were stratified by bulbar- and spinal-disease-onset ALS. Despite controls scoring significantly higher than participants with ALS on executive measures of working memory (*p* = 0.01) and verbal fluency (*p* = 0.001), collectively, patients did not significantly differ on the RMET when compared to the age-, gender-, education-, and IQ-matched TCs (*p* = 0.571). There was, however, a significant difference between disease subtypes, such that participants with bulbar-onset ALS displayed greater mentalising deficits than the spinal-onset group (*p* = 0.001). Nonsignificant results were noted between participants with ALS and TCs on a Judgement of Preference Task employed to examine further affective processes (*p* = 0.057). 

Similar results were found by Burke et al. [43]: for them, TCs did not significantly differ from participants with ALS with the ‘No Cognitive Abnormalities’ (NCA) cognitive phenotype (*p* = 0.157) on the RMET, though TCs did score higher than those with a ‘single executive deficit’ (*p* = 0.002). This difference was not observed when the scores of ALS-NCA were compared to participants with a ‘single executive deficit’ (*p* = 0.118). Both the TC and ALS-NCA performed better than participants with ALS with ‘multi-domain executive impairment’ (*p* ≤ 0.001, respectively).

The second most common form of MND is X-linked spinal and bulbar muscular atrophy (SBMA), also known as Kennedy’s disease (KD: Kennedy, Alter, and Sung [55]. Di Rosa and colleagues [38] conducted a study of ToM, empathy, and neuropsychological functioning in a study of 20 participants with SBMA and 18 age- and education-matched controls. While significant differences were found between patient and TC groups on the Social Faux Pas Test (*p* = 0.02), there were no significant differences observed on the RMET. Interestingly, both SBMA and TC groups scored relatively low on the RMET, with an average SBMA score of 21.78 ± 4.53, compared to TCs, with 22.42 ± 5.43 (*p* = 0.29). Di Rosa et al. [38] reported a deficit in cognitive ToM in the context of an otherwise intact cognitive profile among participants with SBMA.

The studies included in the meta-analysis for the MND group included four studies with ALS [15,39,42,43] and one SBMA study [38]. Of the 383 participants in this sample, 218 were diagnosed with MND and 165 were TCs. The results revealed a significant yet small effect size (SMD, −0.41; 95% CI, −0.61 to −0.20; *p* < 0.05) of MND on RMET performance. There was also a substantial level of heterogeneity within this sample (χ² = 12.25; I² = 67%), which is to be expected given the cognitive, behavioural, and disease-specific phenotypes associated with MND. 

#### 3.5.5. Multiple Sclerosis (MS)

Dulau et al. [44] assessed social cognition in a sample of patients with MS and included mixed-group comparisons according to disease phenotype, i.e., relapsing–remitting MS (RRMS), secondary progressive MS (SPMS), and primary progressive MS (PPMS). The study used the ‘Bordeaux Social Cognition Evaluation Protocol’ (PECS-B) to assess multiple social cognitive domains, such as facial emotion recognition, ToM, emotional awareness, and cognitive and affective alexithymia. Of their sample, 43% of participants in the MS group were impaired on one social cognition test, with 20% impaired on two or more social cognition tests; this was similar across all three phenotypes (i.e., 20%). The performance on the RMET differed significantly between the total MS group when compared to age-, gender-, and education-matched TCs (*p* = 0.003). In addition, the performance on the RMET was positively and significantly correlated with executive function (*r*^2^ = 0.36; *p* < 0.01), working memory (*r* = 0.54; *p* < 0.001), attention (*r* = 0.42; *p* < 0.01), processing speed (*r* = 0.27; *p* < 0.05), and episodic memory (*r =* 0.43; *p* < 0.05). Despite differences between the overall MS group and TCs, the performance on the RMET did not significantly differ between MS-phenotype groups. The proportion of ToM impairment in the MS group as a whole was 28% for ToM (inclusive of the RMET, Attribution of Intention task, and Faux Pas test), over and above facial recognition (10%), emotional awareness (15%), and alexithymia (12%).

Raimo et al. [14] found participants with MS to be impaired on both cognitive and affective ToM, as assessed using four ToM tasks of both verbal and non-verbal modality (F = 40.86; *p* < 0.0001), one of which was the RMET (F = 16.87; *p* < 0.002). The study compared a mixed sample of participants with MS to age-, gender-, and education-matched TCs and included substantially more RRMS (*n* = 36) than SPMS (*n* = 2) or PPMS (*n* = 2) participants. A correlation analysis revealed that RMET performance was not significantly related to other ToM tasks (i.e., Advanced Test of ToM, *r* = 0.2, *p* = 0.21; Emotion-Attribution Task, *r* = 0.28; *p* = 0.09). For both cohorts individually, the RMET did correlate with measures of executive function, i.e., the Symbol Digit Modalities Test (*r*= 0.58; *p* < 0.001) and Stroop test (*r* = 0.64; *p* = < 0.001). There was a significant negative correlation between the RMET and a measure of alexithymia, i.e., the TAS-20, (*r* = −0.33; *p* = < 0.01). 

Pitteri et al. [46] examined social cognitive performance in participants with RRMS in the absence of generalized cognitive impairment, with structural neuroimaging of the amygdala. The study found that participants with RRMS had significantly fewer correct responses than TCs on the RMET (*p* < 0.001), with the bilateral amygdala cortical lesion volume being a significant predictor of task performance (*r* = −0.611; *p* < 0.001). Similar results were also observed using a facial-affect recognition test.

Realmuto et al. [47] evaluated cognitive and social cognitive outcomes in an RRMS group. Most participants with RRMS (77.6%) showed significant cognitive impairment, mainly those pertaining to executive functions, with their performance on the RMET not being significantly different when compared to that of a TC group. As the RMET was not the primary outcome in this study, the specific outcomes for the observed findings were not included, nor were they available upon request. Consequently, the aforementioned study could not be included in the meta-analysis. 

Bisecco et al. [50] explored the association between resting-state functional connectivity and measures of social cognition. Participants with MS were reported to be cognitively intact. Significant group differences were found on a ToM Picture Sequencing Task, but not for the RMET. 

Four studies in the meta-analysis examined RMET performance among participants with MS [14,44,46,50]. There were 172 participants with MS and 168 matched TCs, and the analysis revealed that the MS cohort performed significantly worse than the TCs on the RMET (SMD, −0.80; 95% CI, −1.02 to −0.58; *p* < 0.01). The analysis further showed low levels of heterogeneity within this sample (χ^2^ = 3.23; I^2^ = 7%)

#### 3.5.6. Parkinson’s Disease

Overall, the six PD studies included in the present systematic review showed strong evidence of a social cognitive impairment. However, there was also substantial variability among the selected studies, suggesting the need for better and continued research on mentalising abilities in PD populations. The first of these [10] compared RMET performance between a group of non-demented participants with PD and those of a TC group. The findings revealed that the participants with PD scored significantly lower than the TCs. The authors also performed a correlation analysis and showed that neuropsychological test scores, disease duration, disease severity, depressive symptoms, and health-related quality of life were not significant contributors to the impaired ToM performance observed among the PD group.

Poletti and colleagues [18] investigated the impact of PD disease severity on mentalising performance by recruiting participants at both early and moderate stages of PD, along with a group of age-matched TCs. The results showed that when performance on the RMET was analysed according to disease stage (i.e., early and moderate), TCs significantly outperformed both PD groups (both *p* < 0.01), and participants with early PD outperformed the moderate PD group (*p* = 0.01). Similar to the Bodden et al.’s [10] study, these authors also examined the impact of other potential sources of variability in their sample. Here, the lower RMET performance shown among both PD groups combined was not significantly altered when controlling for age, education, the MMSE, BDI, Frontal Assessment Battery (FAB), or the Montreal Cognitive Assessment (MoCA). However, significant ToM differences which were shown between early- and moderate-stage PD groups were not significant when these factors were covaried.

Enrici et al. [16] also found a significant mentalising deficit in participants with both early and moderate PD (mean time since onset, 10.56 ± 3.88 years; mean Hoehn and Yahr (H&Y) scores of 2.81 ± 0.86) when compared to the TC group on the RMET. Similar to the results of Bodden et al. [10], deficits in RMET performance in the PD group remained when factors such as disease severity, duration of illness, and depressive symptomatology were covaried for, alongside an additional examination of dopamine therapy, cognitive status, executive function, anxiety, and apathy. A measure of facial recognition accuracy was also extracted as a control task by asking all participants to judge the gender of the face in an additional trial of target stimuli.

A follow-up paper, Reference [45], examined the effects of deep brain stimulation (DBS) to the subthalamic nucleus (STN) on social cognition in participants with PD. Participants with PD were divided into two groups (STN-DBS and dopaminergic replacement therapy) and compared to TCs. There was a significant difference between the PD and TC groups when compared on performance on the RMET, although no within group differences were found when PD groups were compared. Enrici et al. [45] suggested that participants with PD undergoing dopaminergic replacement therapy or STN-DBS experience deficits in social cognitive domains, but that STN-DBS does not negatively impact social cognitive performance in isolation. As cognitive impairment is contraindicated for an individual proceeding for DBS at the outset, these findings may have large clinical impact, which is discussed below.

One study [48] presented conflicting evidence whereby mentalising abilities were preserved in participants with early PD diagnosis when their performance on the RMET was compared to TCs (*p* = 0.85). The study analysed data from participants with PD with mild-to-moderate PD, according to the H&Y criteria. Romosan et al. [48] reported that the MoCA total score significantly predicted mentalising performance, suggesting that impaired cognitive functioning was significantly associated with impaired performance on the RMET. Moreover, a multiple regression analysis containing three cognitive domains (specifically, attention, executive function, and visuospatial abilities) explained 64% of the variance and was significantly associated with performance on the RMET. The findings revealed a significant indirect effect of PD on ToM through cognitive status (effect estimate, −4.38; 95% CI, −6.28; −2.67), and cognitive performance appeared to mediate the relationship between PD and affective ToM through the combined effect of attention, executive function, and visuospatial abilities (total effect, −3.63; *p* = 0.001; 95% CI, −5.74; −1.51). 

Similar to previous studies, Reference [51] investigated performance on the RMET and MMSE, alongside topographical and neurochemical bases, using multi-tracer molecular neuroimaging and quantitative electroencephalography. Their cohort consisted of 30 individuals with drug-naïve de novo PD (*n* = 30) and matched TCs (*n* = 60), and they also investigated depression. In this study, there was no significant difference in the participants’ demographic information (age, education, or gender), cognitive status (based on MMSE), or self-reported depression. There was, however, a statistically significant lower RMET performance observed in the PD group compared to TCs (20.7 ± 5.5 vs. 27.5 ± 3.0, respectively; *p* = 0.001). Reference [51] further investigated the relationship between positive, negative, and neutral outcomes on the RMET. Statistically significant negative correlations between both positive and negative stimuli on the RMET, both cumulatively and individually, were observed in relation to the thalamus on the less-affected brain hemisphere, while controlling specifically for background metabolic uptake on metabolic scanning. This study further shows a direct association between RMET performance and cortical metabolic levels in the superior temporal gyrus and the insula, as well as with a higher subcortical serotoninergic tone.

Considering the meta-analysis (Figure 3), a large effect was observed (SMD, −0.95; 95% CI, −1.16 to −0.74; *p* < 0.001), indicating that the RMET performance was significantly lower in PD as compared with their TC groups. However, there were also high levels of heterogeneity reported in this analysis (χ^2^ = 13.89; I^2^ = 71%).

## 4. Discussion

This study’s main findings show that performance on the RMET is largely impacted and significantly lower in neurodegenerative-condition cohorts compared to matched TC groups. Firstly, mentalising deficits (ability to interpret the mental state of others) were evident among participants with mild-to-moderate AD when compared to age- and education-matched TCs (mean ± SD; 24.44 ± 4.32; *p* < 0.04; [36]). Although Heitz et al. [41] found no significant difference in performance on the RMET when participants with probable/early AD were compared to a TC group, the meta-analysis showed that AD groups performed significantly worse on the RMET when combined, with a moderate effect size (SMD, −0.70; 95% CI, −1.21 to −0.18; *p* < 0.01). Significant mentalising deficits were also evident from comparisons between HD groups and TCs included in this review, with results indicative of a mentalising impairment irrespective of disease stage, e.g., premanifest, mild, or moderate [35,37,40,49]. Varying results were found when mixed samples of patients with MS were compared to TCs. Dulau et al. [44] and Raimo et al. [14] revealed a significant difference in performance on the RMET when patients with MS were compared to matched TCs. Reference [46] found that participants with RRMS had significantly fewer correct responses than TCs on the RMET (*p* <0.001); however, these findings were not replicated by Realmuto et al. [47], in which performance on the RMET did not significantly differ when those in the RRMS group were compared to those in a TC group. The meta-analysis revealed that the MS cohort performed significantly worse than TCs on the RMET, with a moderate effect size found (SMD, −0.80; 95%, CI −1.02 to −0.58; *p* < 0.01). Lastly, few included studies did not yield significant differences in RMET performance, and this was particularly evident among, but not limited to, the MND cohorts. Participants with ALS did not score significantly different on the RMET when compared to TCs [15,39,42], with similar results observed when participants with SBMA were compared to TCs [38]. 

The overall objective of this study was to carry out a systematic review outlining performance on the RMET when people with neurodegenerative conditions were compared to TCs. In doing so, the primary aim was to identify whether people with neurodegenerative conditions perform differently from TCs on this measure. A second aim was to collect and collate the performance data from the TC cohorts and subsequently create a normative sample of reference data for which future groups’ performances or an individual’s performance on the RMET could be compared against. Consequently, data from N = 601 TCs across eight countries were compiled. Together, these findings highlight the need for a comprehensive clinical assessment of social cognitive processes in neurodegenerative populations and, therefore, should be considered for inclusion in the routine assessment of cognitive function. Assessments should not be limited to mentalising tasks; instead, it is recommended that clinical batteries incorporate other forms of measurement, such as, by way of example, the Social Faux pas test, Attribution of Intention, and Judgement of Preference tasks, in order to capture a full representation of one’s overall social cognitive abilities. It may also be beneficial to include routine social cognitive assessments throughout the disease progression, as degeneration at later disease stages may affect the function of different social cognitive domains and thus affect a patients’ ability to accurately complete assessments.

The RMET is preferable and reliable for use in neurodegenerative cohorts, as it is multimodal, meaning that items can be verbally responded to or can be pointed to, or eye-gaze technology can be incorporated to indicate the desired preference. Given this multimodality, it can be used longitudinally which may be important for repeat assessments in measuring disease progression over time, specifically where there is a motor component to the condition. There are also short forms of the RMET which have been used with neurodegenerative cohorts and show excellent psychometric properties (MND [26]), which have been validated against large TC groups [27], and others also consider supplementary scoring profiles, i.e., outcomes on positive, negative, and neutral stimuli [29]. The ecological validity of the RMET can be seen as one limitation of the measure, such that interactions with people in daily life require the interpretation of moving expressions as opposed to static ocular images. Despite the high level of heterogeneity both between and within groups, the overall effect size and difference between patients and TCs were significant.

### 4.1. Summary of Main Findings

The meta-analysis component of this review considered the overall comparison between people with a neurodegenerative condition when compared to a typical matched control cohort. Overall, as shown in Figure 2, this study shows that performance on the RMET is significantly lower among people with a neurodegenerative condition than TCs. When stratified by clinical group (Figure 3), DLB groups could not be included due to the single study inclusion for these cohorts. There was, however, a consistently lower performance on the RMET reported for the AD, HD, MS, MND, and PD groups. 

The cohort with HD represented the largest effect size, and significant between-group differences on the RMET were observed, regardless of disease stage or the presence and severity of executive dysfunction [35,37,40,49]. These findings are consistent with the known cognitive profile associated with HD [56], highlighting that disease stage and executive deficits are linked to errors in mental-state attribution but are not the sole cause [57]. As with many of the neurodegenerative cohorts, prospective longitudinal follow-up studies are required to further investigate these relationships and the intra-individual variability on measures of cognitive function. Overall, the results indicate that participants with HD display a lower performance on the RMET relative to TCs. 

Participants with PD exhibited the next largest effect size when performance on the RMET was compared between groups. RMET-specific impairments were reported across the early-to-moderate disease stages [10,16,45]. While not all studies found a significant difference between PD cohorts and TCs [48], RMET-specific deficits have been found in the absence of other cognitive impairments, depression, and/or medication effects [51]. Similar to the HD group, these data would suggest that social cognition, specifically mentalising, may be impaired in PD across disease stages, independent of other cognitive functions. However, there was also substantial variability among the selected studies, suggesting the need for better and continued research on mentalising abilities in PD populations. 

The importance of an intra-individual assessment of cognitive function, as well as fractionating cognitive networks, is highlighted well through the MS cohort. Many studies predominantly report RRMS cohorts with significant mentalising impairment in the absence of non-social cognitive impairment observed [14,44,46,50]. As expected with MS, lesion localization and lesion volume are significant predictors of performance on measures of social cognition [46], as some studies report executive impairment but not social cognitive or mentalising impairments [47]. Deficits in social cognition among participants with MS have been reported to involve global and focal grey matter atrophy, in addition to microstructural white matter damage. However, Realmuto et al. [47] did not find significant global and/or grey-matter atrophy or social cognitive impairment. Overall, this pattern of outcomes may be due to differences in lesion location and neural networks associated with the processing of affective and cognitive mental states and involving frontotemporal and frontoparietal white-matter connections [58]. 

The remaining group which significantly differed from the controls within the meta-analysis were the AD cohorts. While Heitz et al. [41] found no significant differences between groups, their cohort included participants in the ‘probable AD’ diagnostic category, and so these participants were likely to be at a very early disease stage. In contrast to this, using the RMET, mentalising deficits have been shown later in the disease stage, with other cognitive functions also impaired, e.g., the Judgement of Preference task [36]. Within the AD group, the deficit observed in mentalising may be due to non-social cognitive decline impacting performance, and further prospective longitudinal studies are recommended to consider this. 

Heitz et al. [41] also recruited a cohort of people with DLB, and so the control cohort for both the AD comparisons and the DLB comparison are the same group. When comparing these groups, there was significant impairment in mentalising reported using the RMET. Participants with DLB also performed worse than controls on a Cognitive Theory of Mind task, as measured by the Social Faux Pas test.

The last cohort for consideration in the systematic review and meta-analysis was the MND group. This group showed the lowest effect size in the meta-analysis when stratified by condition. Both participant groups with ALS and SBMA/Kennedy’s disease were included in the MND cohort; however, stratifying further to an ALS only group did not significantly change the outcome. Many of the studies in this review report the cognitive phenotype of the ALS group to be within the ‘No Cognitive Abnormalities’ grouping. Interestingly, while both Girardi et al. [15] and Di Rosa et al. [38] found no statistically significant difference in performance on the RMET, they did report a significant difference when the groups were compared on the Judgement of Preference task and Social Faux Pas test, respectively, which is a similar cognitive-dissociation finding to that of Laisney et al. [36]. This further highlights the granular nature of an affective theory of mind processes and highlights the need for comprehensive intra-individual testing. Regarding the RMET and the remaining ALS groups, there were no further significant between-group differences noted [39,42], but a significant stepwise impairment was noted by Burke et al. [43] when the RMET performance was stratified by severity of executive dysfunction. While the MND cohort as a whole presented with impaired outcomes on the RMET, overall, the ALS phenotype with no cognitive abnormalities does not appear to present specifically with mentalising deficits, as measured using the RMET, in the absence of executive and non-social cognitive dysfunction. 

### 4.2. Strengths, Limitations, and Future Considerations

The inclusion criteria required clinical cohorts to have matched TCs. While this is a strength in terms of developing meta-norms and conducting a meta-analysis, it excludes studies with no control group. Similarly, this review considered only the full 36-item version of the RMET, rather than shortened or altered versions. Collectively, this is likely to be a reason for the low inclusion rates for some neurodegenerative conditions where social cognitive decline is a main feature, e.g., bvFTD, where the RMET has been used; however, in some studies, no control group was recruited, or a modified version of the RMET was adopted [59,60,61,62].

There are both strengths and limitations related to systematically reviewing a single measure—in this case, performance on the RMET—over and above more broadly systematically reviewing the concept of mentalising. By virtue of this review, specifically investigating performance on the full version of RMET as a primary measure, this review does not aim to provide a full overview of social cognition in neurodegenerative conditions; however, other systematic reviews of this are available [24]. Notwithstanding, by systematically reviewing and completing a meta-analysis of the full RMET across all neurodegenerative conditions, in a time-locked review, this study highlights the clinical sensitivity of the RMET as a sensitive marker of cognitive function that is specific to mentalising.

This review demonstrates the importance of assessing social cognition in neurodegenerative cohorts and highlights the need for greater classification, categorisation, and theoretical understanding of the cognitive neuroscience, which underpins social cognitive performance. There are limitations within the original studies which then translate to this review, in that no consistent measure of function was used in order to determine a baseline or equivalency of function, i.e., IQ scores for each participant group. While this means that a portion of the variance observed is likely to be due to sample variance, it speaks to future research to include outcome measures in their design, which would allow reviews to further consider the equivalency of samples. To date, there is no consensus cognitive model of social cognition, and future research should aim to further synthesise and develop what is known in order to consider this. 

## 5. Conclusions

These findings highlight the importance of including a measure of mentalising in standardised cognitive batteries, and this review provides adult-based meta-normative data for the 36-item version of the RMET (mean raw score, 25.20 ± 3.66; average percentage correct, 70% ± 10.16%). Considering the findings from this review in total, these results suggest that the RMET can detect changes in social cognition in people with neurodegenerative conditions and is dissociable from other cognitive and social cognitive processes. However, due to the heterogeneous nature of the conditions investigated here and the homogeneous finding that the RMET is impaired when compared to matched typical controls, additional and further assessment of social cognitive processes is likely warranted to fully elucidate the nuance of social cognitive impairment within these conditions. In line with this, further work is also needed within the area to investigate the intra-individual variability on measures of social cognition and specifically how they relate to measures of cognitive function. This review highlights that social cognition as measured by the RMET can be impaired alongside and in the absence of poor general cognitive status (MMSE; MoCA), specific executive dysfunction (verbal fluency and Trail-Making Test), language impairment (semantic fluency), and other measures of social cognition (social faux pas test), but not exclusively. 

## Figures and Tables

**Table 1:** A summary of included studies.

Authors (Year)	Country	Design	Sample, *n* (% Female)	Age in Years, Mean ± SD	Mean Years of Education ± SD	Mean Time Since Disease Onset ± SD (Time)	Mean Disease Duration ± SD (Years)
Bodden et al. [10] *	Germany	Cross-sectional	21 PD (29%) 21 TC (29%)	63.7 ± 10 PD 58.5 ± 10.2 TC	14.6 ± 3.0 PD 14.8 ± 2.9 TC	-	5.1 ± 2.8
Girardi et al. [15]	Scotland	Cross-sectional	14 ALS (29%) 20 TC (25%)	57.4 ± 16 ALS 54.8 ± 11.5 TC	14.4 ± 5.4 ALS 15.1 ± 3.2 TC	38.1 ± 29.6 (months)	-
Allain et al. [35]	France	Cross-sectional	18 HD (44%) 18 TC (39%)	50.7 ± 8.8 HD 47.7 ± 8.9 TC	11.8 ± 2.5 HD 11.1 ± 2.4 TC	-	4.5 ± 2.9
Laisney et al. [36]	France	Cross-sectional	16 AD (69%) 15 TC (80%)	78.1 ± 2.6 AD 76.4 ± 3.2 TC	7.7 ± 2.5 AD 7.5 ± 1.6 TC	-	-
Poletti et al. [18]	Italy	Cross-sectional	35 PD (37%) 35 TC (57%)	68.5 ± 6.7 PD 67 ± 10 TC	9.1 ± 3.9 PD 10.8 ± 3.8 TC	-	6.31 ± 4
Eddy et al. [37] *	UK	Cross-sectional	30 HD (40%) 20 TC (50%)	54.9 HD 47.3 TC	13 ± 2.3 HD 13.9 ± 2.1 TC	-	7.03 ± 4.9
Di rosa et al. [38]	Italy	Cross-sectional	20 SBMA (0%) 18 TC (0%)	55.4 ± 9.4 SBMA 55.1 ± 10.3 TC	11.4 ± 4.3 SBMA 10.3 ± 3.9 TC	-	-
Jelsone-swain et al. [39]	USA	Cross-sectional	19 ALS (32%) 18 TC (39%)	57.2 ± 8.7 ALS 59.9 ± 8.6 TC	13.6 ± 2.1 ALS 16.3 ± 2.4 TC	47 ± 33.7 (months)	-
Enrici et al. [16]	Italy	Cross-sectional	32 PD (47%) 25 TC (56%)	58 ± 7.2 PD 56.3 ± 6.4 TC	9.5 ± 4 PD 10.8 ±3.8 TC	-	10.6 ± 3.9
Eddy et al. [40] *	UK	Cross-sectional	20 HD (70%) 26 TC (69%)	45 ± 14 HD 45.7 ± 14.4 TC	14 ± 2.5 HD 14 ± 1.9 TC	-	-
Heitz et al. [41]	France	Longitudinal	33 DLB (52%) 15 AD (47%) 16 TC (56%)	68 ± 8.4 DLB 70.9 ± 11.1 AD 68.3 ± 10.5 TC	12.4 ± 3.2 DLB 13.5 ± 3.6 AD 11.8 ± 3.2 TC		4.6 ± 4.2 DLB 3.6 ± 1.8 AD
Burke et al. [42]	Ireland	Cross-sectional	106 ALS (28%) 50 TC (40%)	60.4 ±10.8 ALS 61.4 ±9.1 TC	12.8 ± 3.3 ALS 12.7 ± 2.9 TC	-	-
Burke et al. [43]	Ireland	Cross-sectional	59 ALS (37%) (bulbar *n* = 20 (65%), spinal *n* = 39 (23%) 59 TC (56%)	66.1 ± 8.8 bulbar-onset ALS 61.2 ± 9.1 spinal onset ALS 64.7 ± 9 TC	12.40 ± 2.58 bulbar-onset ALS 13.46 years ± 3.49 spinal onset ALS 12.64 ± 3.07 TC	10.8 ± 9.2 bulbar-onset ALS (months) 14.4 ± 12.7 spinal onset ALS (months)	-
Dulau et al. [44]	France	Longitudinal	60 MS (NR) 65 TC (NR)	46.5 ± 10.6 MS (40.1 ± 10.4 RRMS; 54.8 ± 11.5 PPMS; 51 ± 7.3 SPMS) 43.2 ± 9.3 TC	12.9 ± 3.3 MS (14.2 ± 3.7 RRMS; 10.9 ± 4.6 PPMS; 12.5 ± 5.3 SPMS) 12.5 ± 2.8 TC	-	14.4 ± 9.4 MS (11.5 ± 5.6 RRMS; 10 ± 6.5 PPMS; 18.8 ± 10 SPMS)
Enrici et al. [45]	Italy	Cross-sectional	20 DRT-PD (50%) 18 STN-DBS-PD (50%) 20 TC (50%)	59.8 ± 5.8 DRT-PD 60.9 ± 6.3 STN-DBS-PD 60 ± 7.5 TC	7.7 ± 2.5 DRT-PD 7.4 ± 3.9 STN-DBS-PD 9.4 ± 3.3 TC	-	11.5 ± 3 DRT-PD 12.6 ± 3 STN-DBS-PD
Raimo et al. [14]	Italy	Cross-sectional	40 MS (73%) 40 TC (78%)	40.6 ± 11.5 MS 40.2 ± 11.4 TC	13.1 ± 3.8 MS 13 ± 3.6 TC	-	8.2 ± 7.5
Pitteri et al. [46]	Italy	Cross-sectional	31 RRMS (77%) 38 TC (74%)	36.3 ± 7.6 RRMS 37.1 ± 8.9 TC	13.4 ± 3.4 RRMS 14.6 ± 3.4 TC		7 ± 4.5
Realmuto et al. [47] *	Italy	Cross-sectional	45 RRMS (69%) 45 TC (71%)	34.2 ± 7.7 RRMS 33 ± 7.7 TC	RRMS 13.4 ± 2.6 TC 13.3 ± 3.1		9.7 ± 6.2
Romosan et al. [48]	Romania	Cross-sectional	65 PD (42%) 51 TC (39%)	58.1 ± 5.3 PD 56.5 ± 6 TC	NR		6.4 ± 3.2
Bayliss et al. [49]	Spain	Cross-sectional	12 HD (67%) 12 relatives (58%) (spouse or gene-negative relative) 12 TC (75%)	Median and IQR: 42.7 (11.3) HD; 44.7 (22.3) HD relatives; 37.1 (15.8) TC	Median and IQR: 16.0 (5.0) HD 16 (6.2) HD relatives 14.5 (7.5) TC	-	-
Bisecco et al. [50]	Italy	Cross-sectional	41 MS (66%) 25 TC (72%)	34.2 ± 10.3 MS 37.8 ± 12 TC	NR	-	8.8 ± 8.2
Orso et al. [51]	Italy	Cross-sectional	30 PD (36%) 60 TC (32%)	73.39 ±8.93 70.1 ± 10.9	11.0 ±3.54 PD 9.5 ±5.4 TC	-	-

Note: SD = standard deviation; PD = Parkinson’s disease; TC = typical control; ALS = amyotrophic lateral sclerosis; HD = Huntington’s disease; AD = Alzheimer’s disease; SBMA = spinal–bulbar muscular atrophy; DLB = Lewy body dementia; MS = multiple sclerosis; RRMS = relapsing–remitting multiple sclerosis; PPMS = primary progressive multiple sclerosis; SPMS = secondary progressive multiple sclerosis; DRT-PD = dopamine-replacement therapy in Parkinson’s disease; STN-DBS-PD = subthalamic-nucleus deep brain stimulation in Parkinson’s disease; IQR = interquartile range. * Study not included in meta-analysis due to missing or unavailable data.

**Table 2:** A breakdown of the methodological quality of each included study.

Study	Title and Abstract	Introduction and Aims	Method and Data	Sampling	Data Analysis	Ethics and Bias	Findings and Results	Transferability/Generalizability	Implications/ Usefulness	Overall
Bodden et al. [10] *	4	4	4	4	4	4	4	3	4	35
Girardi et al. [15]	4	4	3	4	3	4	4	4	4	34
Allain et al. [35]	4	4	4	4	4	4	4	4	4	36
Laisney et al. [36]	4	4	3	3	4	1	4	3	3	29
Poletti et al. [18]	4	2	3	2	4	4	4	2	3	28
Eddy et al. [37] *	4	3	4	4	3	4	4	4	4	34
Di rosa et al. [38]	3	4	4	4	4	1	4	4	3	31
Jelsone-swain et al. [39]	4	4	4	3	3	4	4	4	3	33
Enrici et al. [16]	4	4	4	4	4	4	4	4	4	36
Eddy et al. [40] *	4	4	4	4	4	4	4	4	4	36
Heitz et al. [41]	4	4	4	4	4	4	4	4	4	36
Burke et al. [42]	4	3	4	4	4	4	4	4	4	35
Burke et al. [43]	4	4	4	4	4	4	4	4	4	36
Dulau et al. [44]	3	3	3	4	4	4	4	4	3	32
Enrici et al. [45]	4	4	4	4	4	4	4	4	3	35
Raimo et al. [14]	4	4	4	4	4	1	4	4	4	33
Pitteri et al. [46]	4	3	4	4	4	4	4	4	4	35
Realmuto et al. [47] *	4	3	3	4	4	1	3	4	3	29
Romosan et al. [48]	4	4	4	4	4	4	4	4	3	35
Bayliss et al. [49]	4	2	3	3	4	4	3	3	3	29
Bisecco et al. [50]	3	4	4	4	4	4	3	4	4	34
Orso et al. [51]	3	3	4	4	4	4	4	4	2	32
Average	3.81	3.54	3.72	3.77	3.86	3.45	3.86	3.77	3.5	33.31

Note: * Study not included in meta-analysis due to missing or unavailable data. Score range (1–4).

## Data Availability

No new data were created or analyzed in this study. Data sharing is not applicable to this article.

## Supplementary Materials

The following supporting information can be downloaded at https://www.mdpi.com/article/10.3390/brainsci13091268/s1, Table S1: Overview of neurodegenerative disease characteristics.
