# Supplementary material for: A 20-Year Systematic Review of the ‘Reading the Mind in the Eyes’ Test across Neurodegenerative Conditions
Source: Brain Sci. 2023 Aug 31;13(9):1268. doi: 10.3390/brainsci13091268 (PMC10526136; doi:10.3390/brainsci13091268)
Supplement: Supplementary file 1 [file brainsci-13-01268-s001.zip › brainsci-2575182-supplementary.pdf]

Supplementary Table S1. Overview of neurodegenerative disease characteristics.

Disease	Neuropsychological presentation	Main neuropathology	Protein aggregate(s)	Main anatomical vulnerability
Alzheimer's disease	Episodic memory[1]<br>Language skills[1]<br>Behaviour change[1]<br>Orientational difficulties[2]<br>Social behaviour change[2]<br>Emotion perception [3]<br>ToM[2]	Amyloid angiopathy[4]<br>Neuritic and amyloid plaques[4]<br>Neuropil threads[4]<br>Neurofibrillary tangles[4]	Aβ[4]<br>3R + 4R tau[4]	Frontal and temporal lobes[4]<br>Limbic structures[4]<br>Basal forebrain[4]
Behavioural variant frontal temporal dementia	Executive dysfunction[5]<br>Personality change[6]<br>Social comportment disorder[5]<br>Reduced social competencies[6]<br>ToM[7]	Asymmetric white and grey matter degeneration[5]<br>Neuronal cytoplasmic inclusions[4]	Tau[8]<br>Straight filaments PHF[8]<br>TDP-43[4]	Right hemisphere[5]<br>Ventral frontal regions[5]<br>Fronto-polar cortex[9]

Dementia with lewy bodies	Attention difficulties[10]<br><br>Executive dysfunction[10]<br><br>Episodic verbal memory[10]<br><br>Apathy<br><br>ToM	Lewy bodies[4]<br><br>Lew neurites[4]	α-synuclein [11]	Cerebral cortex[4]<br><br>Amygdala[4]<br><br>Hippocampus[4]<br><br>Substantia nigra[4]
Huntington's disease	Motor decline[12]<br><br>Executive function[13]<br><br>Working memory[14]<br><br>Processing speed[14]<br><br>Emotion recognition[14]	CAG expansion in the huntingtin gene[12]	Huntingdon nuclear inclusions[8]	Caudate and putamen gray matter change[15]<br><br>Striatum[15]<br><br>Corpus callosum[15]
Mild cognitive impairment	Isolated cognitive deficit in the absence of dementia[16]<br><br>Isolated deficit of memory = aMCI[16]<br><br>Executive dysfunction[16]<br><br>ToM[16]	Precursor to AD development[16]	Tau[17]	Prefrontal lobe[16]<br><br>Medial temporal lobe[16]<br><br>Hippocampus[17]<br><br>Amygdala[17]
Motor neuron disease –	Executive dysfunction[13]<br><br>Behaviour change[13]	Upper and lower motor neuron loss[4]	TDP-43[4]	Motor cortex[4]<br><br>Spinal cord and brainstem motor neurons[4]

Amyotrophic lateral sclerosis	Apathy[13]	Neuronal inclusions[4]<br>Astrocytic hyaline inclusion[4]		Orbitofrontal cortex[13]<br>Prefrontal cortex[13]<br>Anterior cingulate[13]
Motor neuron disease – Spinal bulbar muscular atrophy	Weakening of bulbar, facial, and limb muscles[18]<br>Attention difficulties[19]<br>Executive dysfunction[19]<br>Verbal memory[20]<br>Communication[18]	Trinucleotide (CAG) repeat expansion in the first exon of the androgen receptor gene[21]	AR[18]	Primary motor cortex[18]<br>Frontal lobe[18]<br>Cerebellum[18]<br>Brainstem[18]
Multiple Sclerosis	Motor decline[22]<br>Executive dysfunction[23]<br>ToM[24]<br>Emotion perception[25]	Demyelination[26]<br>Axonal damage and loss[26]<br>Reactive gliosis[26]<br>Inflammation[27]	Myelin basic protein[28]	CNS[26]
Parkinson's Disease	Motor disturbance[29] (i.e., rigidity, postural instability)<br>Verbal learning memory[29]	Neuronal degeneration[30]<br>Dopaminergic denervation[30]	α-synuclein[8]	Substantia nigra[30]<br>Basal ganglia[29]<br>Limbic system[29]

	Visuospatial processing[29]
	Emotional dysfunction[29]

Note. ToM = Theory of mind; CNS = Central nervous system; [1]Agrawal & Biswas, 2015, [2]Kessels *et al*. 2021, [3]Torres Mendonça De Melo Fádel *et al*. 2019, [4]Dugger & Dickson, 2017, [5]Irwin *et al*. 2018, [6]Rascovsky *et al*. 2011, [7]Adenzato *et al*. 2010, [8]Relja, 2004, [9]Torralva *et al*. 2009, [10]Hansen *et al*. 2021, [11]Schumacker *et al*. 2021, [12]Scahill *et al.* 2020, [13]Burke *et al*. 2017, [14]Labuschagne *et al.* 2016, [15]McColgan *et al*. 2018, [16]Poletti & Bonuccelli, 2013, [17]Moreau *et al*. 2015, [18]Di Rosa *et al*. 2015, [19]Soukup *et al*. 2009, [20]Guidetti *et al*. 1996, [21]Spada *et al*. 1991, [22]Lin *et al*. 2020, [23]Giazkoulidou *et al*. 2019, [24]Mike *et al*. 2013, [25]Radlak *et al*. 2021, [26]Reynolds *et al.* 2011, [27]Sobel & Moore, 2008, [28]Krugmann *et al*. 2020, [29] Parkash *et al.* 2016, [30] Dickson, 2018
